# Muscular Power

**Published:** 1852-10

**Authors:** 


*Muscular Power.*—It is a matter of most interesting thought that size and construction seem to have but litttle influence in regulating the comparative speed and strength of animated beings. Man has the ability to imitate all motion but that of flight, perhaps from the great number of bones, muscles and ligaments composing the human machine, excelling all other animals in this respect; the head containing 63 bones; the trunk 53; upper extremities 64; and the lower extremities 68; connected together by 434 muscles and 125 to 2 or 300 ligaments, according to the manner of their classification. The heart makes 64 pulsations in a minute; 3840 in an hour; 92,160 in a day; with these complete circulations of his blood in the short space of an hour; this is perhaps the most wonderful phenomenon in the composition of man, the motive power of which may be considered disputed territory, having many theories, supported by many advocates; in all of which, may, most likely be found an addition to the true cause of its propulsion.

The sloth is not by any means a small animal, and yet only capable of moving a few paces a day. Many insects of considerable size are incapable of much power or motion in a given time, and yet others infinitely less, as the lady bird can fly twenty millions of times its own length in less than an hour; a worm can only crawl an inch or two in a minute, whereas an antelope can run a mile in one minute; an elk a mile in seven and a half minutes; the wild mule of Tartary is even greater in speed than either of these. The eagle is said to fly 54 miles in an hour, and some of the species of the falcon being able to reach 250 leagues in the space of sixteen hours, almost the velocity of wind, which travels 60 miles an hour; sound 1142 English feet per second, and light 192,000 miles per second.

